# Against Dataism and for Data Sharing of Big Biomedical and Clinical Data with Research Parasites

**DOI:** 10.3389/fgene.2016.00154

**Published:** 2016-08-31

**Authors:** Frank Emmert-Streib, Matthias Dehmer, Olli Yli-Harja

**Affiliations:** ^1^Predictive Medicine and Analytics Lab, Department of Signal Processing, Tampere University of TechnologyTampere, Finland; ^2^Department of Mechatronics and Biomedical Computer Science, UMITHall in Tyrol, Austria; ^3^College of Computer and Control Engineering, Nankai UniversityTianjin, China; ^4^Computational Systems Biology, Department of Signal Processing, Tampere University of TechnologyTampere, Finland

**Keywords:** data sharing, clinical data, biomedical data, genomics, computational biology

According to the Oxford Dictionaries Online, Medicine is “The science or practice of the diagnosis, treatment, and prevention of disease.” This implies that a patient is in the central focus of the profession and all relevant specializations and subareas are concerned with benefiting a patient's health. In recent years, the analysis of clinical and biomedical data, including high-throughput experiments, has been added to the list of such specializations that make contributions for the greater good. However, the analysis and the reuse of such data is in general difficult and for this reason has been under scrutiny (Ioannidis, 2005; Chalmers and Glasziou, 2009; Ioannidis and Khoury, 2011; Rung and Brazma, 2013; Ioannidis et al., 2015).

With breakthroughs in data production, the integration of unprecedentedly rich data is expected to lead to an enormous impact on basic research and to translate on healthcare, but comes with significant challenges for the practices of analysis, data sharing, and the evaluation of results (Marx, 2013; Fan et al., 2014; Emmert-Streib et al., 2016). Improvements in these areas would undoubtedly make research process more efficient and its results more reliable. An important case is offered by Baggerly and Coombes (2009) who found by the *re-analysis* of various data sets from Potti et al. (2011) fundamental flaws leading ultimately in the discontinuation of three clinical cancer trials. This became known as Duke Saga (Kolata, 2011). It is difficult to quantify their impact on the health of patients but given they even identified erroneous therapeutic interventions based on the work of Dr Potti, it is fair to assume that their work helped even saving the life of patients. Given this contribution and its clearly beneficial impact for patients it is stunning that according to a recent publication by Longo and Drazen (2016) scientists like Keith Baggerly and Kevin Coombes have been pejoratively characterized as “research parasites.”

Regarding regulations for data sharing, a major point made in a series of papers published in the New England Journal of Medicine (NEJM; Drazen, 2016; Longo and Drazen, 2016; Taichman et al., 2016) was that

1. “Those using data collected by others should seek collaboration with those who collected the data” (Taichman et al., 2016)

and

2. “Report the new findings with relevant coauthorship to acknowledge both the group that proposed the new idea and the investigative group that accrued the data that allowed it to be tested” (Longo and Drazen, 2016).

The initial reaction of the computational research community has not been positive (Berger et al., 2016; McNutt, 2016).

We are of the opinion that both suggestions are reasonable as “can rules” if circumstances allow it, however, we think that neither should be mandatory. The reason for this is simple. Let's say a published data set, and by this we mean a data set that had to be made publicly available in order to publish major findings in a journal or an obligation imposed by a funding agency, is re-analyzed. In the following we call the scientists generating the data “experimental party” and the scientists re-analyzing the data “computational party.” There are three possible outcomes. First, no results are found which means nothing needs to be published. Second, results are found and both parties are happy with the conclusions. In this case the results can be published and the experimental party could be offered coauthorship but only if the usual criteria for receiving an authorship are met, requiring a significant contribution *beyond* merely providing the data. Third, results are found but both parties disagree with the conclusions. This is certainly the most interesting outcome that deserves attention and is also the case in the Duke Saga. The problem with requiring to name the experimental party as coauthors could be a conflict of interests preventing a paper even from being submitted to a journal for review. Hence, there would be a leverage one would give to such authors allowing to at least delay such a submission indefinitely. For instance, we could ask ourselves at what time point after the accusation made by Keith Baggerly and Kevin Coombes would Anil Potti have agreed to be a coauthor on the paper in Baggerly and Coombes (2009)? The answer to this question is unknown, however, it is not difficult to see the problems that are implied by such a “must” rule that are clearly not beneficial for the patients enrolled in clinical trials based on flawed benefits.

From the outline of these problems, we suggest the following rules for data sharing:

Mandatory rules:
M1 In the publication of an article re-analyzing published data, add a citation to the original publication(s) of the data.
M2 A possible communication with the experimental party should be acknowledged in the published article.
M3 The code used for re-analyzing the data should be made publicly available.
Optional rule:
O1 If the computational and the experimental parties agree on the research findings declaring no conflict of interest and the experimental party contributes significantly to the re-analysis, both parties should receive authorship.

In addition to this, we consider it obligatory for journals publishing articles to turn out being erroneous that they publish the articles revealing these issues. For instance, Anil Potti had to retract papers published in Nature and Science but the paper by Keith Baggerly and Kevin Coombes wasn't accepted there, instead, it appeared in the Annal of Applied Statistics (Baggerly and Coombes, 2009). This is not acceptable!

The above rules M1–M3 will ensure that it is possible that the re-analysis of data can “disprove what the original investigators had posited” (Longo and Drazen, 2016) because if the initial analysis is wrong this needs to be revealed without any hesitation or qualification.

From a more fundamental point of view the above question of data sharing has an analogy with capitalism. The reason for this is that in capitalism the capital (money) can generate more capital without labor by means of interests. In our case the new capital is data which, according to the rules suggested by Longo and Drazen (2016), Drazen (2016), and Taichman et al. (2016), can generate authorship(s) without contributing to the re-analysis of data ad infimum. As such it would change the way we know science completely. That means the question we need to ask ourselves is do we want a dataism (Lohr, 2015) in science that allows such a monopoly? We are strictly against such a monopoly based on data and for this reason suggested publication rules that prevent this from happening and plead for a data sharing with “research parasites” in the interest of the patients from whom the data originate.

## Author contributions

FE conceived the study. FE, MD, and OY wrote the paper.

## Funding

FE would like to thank TUT for financial support. MD thanks the Austrian Science Funds for supporting this work (project P26142).

### Conflict of interest statement

The authors declare that the research was conducted in the absence of any commercial or financial relationships that could be construed as a potential conflict of interest.
